# Toxicity and Safety Evaluation of Doxorubicin-Loaded Cockleshell-Derived Calcium Carbonate Nanoparticle in Dogs

**DOI:** 10.1155/2018/4848602

**Published:** 2018-06-24

**Authors:** Abubakar Danmaigoro, Gayathri Thevi Selvarajah, Mohd Hezmee Mohd Noor, Rozi Mahmud, Md Zuki Abu Bakar

**Affiliations:** ^1^Department of Veterinary Preclinical Science, Faculty of Veterinary Medicine, Universiti Putra Malaysia, 43400 Serdang, Selangor Darul Ehsan, Malaysia; ^2^Department of Veterinary Anatomy, Faculty of Veterinary Medicine, Usmanu Danfodiyo University, P.M.B. 2346, Sokoto, Nigeria; ^3^Department of Veterinary Clinical Studies, Faculty of Veterinary Medicine, Universiti Putra Malaysia, 43400 Serdang, Selangor Darul Ehsan, Malaysia; ^4^Department of Imaging, Faculty of Medicine and Health Science, Universiti Putra Malaysia, 43400 Serdang, Selangor Darul Ehsan, Malaysia

## Abstract

Doxorubicin (DOX) is a potent anticancer agent with cytotoxic effects which limit its clinical usage. This effect is due to its nonselective nature causing injury to the cells as a result of reactive free oxygen radical's release. Cockleshell-derived calcium carbonate nanoparticle (CS-CaCO_3_NP) is a pH-responsive carrier with targeted delivery potentials. This study aimed at evaluating the toxicity effects of repeated dose administration of DOX-loaded CS-CaCO_3_NP in healthy dogs. Fifteen dogs with an average body weight of 15 kg were randomized equally into 5 groups. Dogs were subjected to 5 doses at every 3-week interval with (i) normal saline, (ii) DOX, 30 mg/m^2^, and the experimental groups: CS-CaCO_3_NP-DOX at (iii) high dose, 50 mg/m^2^, (iv) clinical dose, 30 mg/m^2^, and (v) low dose, 20 mg/m^2^. Radiographs, electrocardiography, and blood samples were collected before every treatment for haematology, serum biochemistry, and cardiac injury assessment. Heart and kidney tissues were harvested after euthanasia for histological and ultrastructural evaluation. The cumulative dose of DOX 150 mg/m^2^ over 15 weeks revealed significant effects on body weight, blood cells, functional enzymes, and cardiac injury biomarkers with alterations in electrocardiogram, myocardium, and renal tissue morphology. However, the dogs given CS-CaCO_3_NP-DOX 150 mg/m^2^ and below did not show any significant change in toxicity biomarker as compared to those given normal saline. The study confirmed the safety of repeated dose administration of CS-CaCO_3_NP-DOX (30 mg/m^2^) for 5 cycles in dogs. This finding offers opportunity to dogs with cancer that might require long-term administration of DOX without adverse effects.

## 1. Introduction

Doxorubicin (DOX) is one of the potent chemotherapeutic agents used in the management of both haematopoietic and solid malignant tumours of different origin [1–3]. In the past two decades, its application by oncologists has been extensively explored, although its continuous usage alone in clinical settings is impeded due to its life-threatening effect on organs such as heart, kidney, and liver [4, 5]. DOX toxicity is generally inclined to the mitochondrial oxidative phosphorylation and calcium ion overload through multiple complex pathways [2, 6]. The mitochondrial, sarcoplasmic reticulum, and nuclear material damages are due to the reactive oxygen radicals released during the biotransformation of DOX to semiquinone [6, 7] and a decrease in ATP synthesis [8]. This mitochondrial and genetic material disruption is believed to be positively correlated with the DOX-induced clinical cardiomyopathies [9, 10].

Several efforts have recently been made to ameliorate the side effects of DOX [2, 11], with the use of lipids and assembled polymers in the delivery of DOX as seen in Doxil™ and Myocet™ which are still associated with mild toxic effects on organs by eliciting proinflammatory cell release [12], with some other formulations shown having an increase in therapeutic efficiency with few setbacks, such as instability and biphasic release kinetic mechanism [13, 14]. However, decrease in toxicity was recorded with liposome conjugated with DOX in a mouse model, although the long-time cumulative effect of the liposome was not documented [15]. In addition, several organic macromolecules have been employed in nanomedicine for drug delivery [16, 17].

Thus, these growing interests in the use of nanocarrier in the delivery of anticancer with little or no accumulative toxic effects on tissue have encouraged the search for naturally occurring biogenic nanomaterials in drug delivery. DOX delivery using nanocarriers is advocated to reduce toxicity and improve therapeutic efficacy [11, 16].

Cockleshell-derived CaCO_3_ nanoparticle (CS-CaCO_3_NP), an inorganic biodegradable drug carrier, has been used to deliver DOX and docetaxel in different experimental studies [18, 19]. Despite the advances in cancer treatment, it still possesses significant threats to oncologists in using DOX in preventing cancer metastasis with its continuous usage hindered by its off-target effects on the proliferating healthy cells [20].

Previous studies indicate that electrocardiography and echocardiography are noninvasive techniques employed in monitoring cardiac injury, which are recommended in the evaluation of dogs undergoing chemotherapy [21, 22]. However, studies have shown that fractional shortening in echocardiography is associated with low specificity as a result of overreliance on preload and postload effect in dogs given DOX at several cyclic schedules [23]. As a result of these, other noninvasive assays are selected for the prediction of DOX-induced cardiotoxicity, such as serum cardiac muscle protein quantification specifically, cardiac troponin, and NT-pro-brain natriuretic peptides [24, 25], which are widely used in preclinical and clinical practices for cardiac injury detection.

The low therapeutic index of DOX has necessitated the search of targeted nanocarrier for DOX delivery in the treatment of solid cancer. CS-CaCO_3_ nanocarrier conjugated with DOX has demonstrated control and slow release in both *in vitro* and *in vivo* studies in dogs [23]. However, there is lack of information on the toxicity and safety profile of the CS-CaCO_3_NP-DOX upon repeated intravenous dose administration in dogs, which is needed to define the maximum tolerable dose (MTD) in dogs, which will serve as a key in attaining therapeutic success. In addition, the use of CS-CaCO_3_NP to deliver DOX is to reduce its off-target effects through its pH-responsive release mechanism. Hence, it is important to note that CS-CaCO_3_ nanocarrier as compared to other commercially available synthetic polymers decomposes slowly in physiological state to produce Ca^2+^ and CO_2_^3−^ and release DOX for therapeutic purposes. The decomposed products could be useful in muscle contraction, bone remodelling, and blood clotting factor, whereas the carbonate component will be excreted through gaseous exchanges. Therefore, this study aimed at evaluating the toxicity and safety effects of repeated dose administration of CS-CaCO_3_NP-DOX in healthy dogs as an alternative carrier from inorganic biogenic source for the delivery of DOX with special interest on the cardiotoxicity and nephrotoxicity induced by DOX using biomarker, histopathology, and ultrastructural morphological changes for assessment.

## 2. Materials and Methods

### 2.1. Materials

Doxorubicin A.D. Mycin (Adriamycin®) 50 mg/25 mL vial was purchased from Boryung Pharmaceutical Co. Ltd., Korea. 0.9% normal saline, Canine NT-proBNP (N-Terminal Pro-Brain Natriuretic Peptide) ELISA kit, and Canine cTn-I/TNNI3 (cardiac troponin I) ELISA kit (E-EL-C0212) were all purchased from the Elabscience, China, and were stored at −20°C. All other chemicals used were of analytical grade and were stored at 25°C.

### 2.2. Synthesis of CS-CaCO_3_NP and Incorporation of DOX into Synthesized CS-CaCO_3_NP

The synthesis, drug loading, and characterization of CS-CaCO_3_NP and CS-CaCO_3_NP-DOX were carried out in accordance with the protocol and procedure developed by Danmaigoro et al. [26]. The synthesized CS-CaCO_3_NP with a higher loading content capacity formulation was selected for DOX delivery.

### 2.3. Repeat Dose Toxicity and Safety Studies in Healthy Dogs

#### 2.3.1. Animal Handling and Ethical Statement

A total of fifteen (15) healthy adult male local dogs (*Canis lupus familiaris*) aged 9–24 months, weighing 15–25 kg, were obtained from the Dewan Bandaraya Kuala Lumpur pound. Ages of the dogs were determined by dental recognition according to Iohara et al. [27] and from the dogs' records. Dogs were cleaned (bathe), detached using Bayticol® (Flumethrin), and dewormed with praziquantel 20 mg/kg. The dogs were kept at 23 ± 1°C for 12-hour light and 12-hour dark cycle with access to feed and *ad libitum* water. All procedures involving animal care and handling according to the guidelines and approval of the Institutional Animal Care and Use Committee (IACUC) (UPM/IACUC/AUP-RO13/2016), Universiti Putra Malaysia, and the recommendation of Canadian Council on Animals Care Guide to the Care and Use of Experimental animals were strictly followed [28, 29]. The dogs were confirmed clinically healthy by haematological evaluation, blood smear for heartworm, and physical screening, with no dogs placed on chemotherapeutic agent during the 2-week period of acclimatization.

#### 2.3.2. Exclusion and Inclusion Selection Criteria in the Study

Dogs with recognizable disease symptoms (vomiting, diarrhoea, cardiac murmur, or arrhythmia), bad temperament, or poor body condition score (1/5) were excluded in the selection and enrolment into the experimental study. However, dogs with normal haematology and serum biochemistry parameters, healthy otherwise, good temperament, and good body condition score were selected and included in the study. Dogs were housed at the Animal Research Facilities (ARF), Faculty of Veterinary Medicine, Universiti Putra Malaysia.

### 2.4. Protocol

The dogs were randomly assigned to 5 groups (*n*=3). Dogs were subjected to slow intravenous infusion (0.9% NaCl) up to 5 doses every 3-week interval with (i) normal saline (0.9% NaCl) (negative control), (ii) DOX 30 mg/m^2^ (positive control), and the experimental groups: CS-CaCO_3_NP-DOX at (iii) high dose, 50 mg/m^2^, (iv) clinical dose, 30 mg/m^2^, and (v) low dose, 20 mg/m^2^, in accordance with the DOX administration schedules. At day 0 (the first day of evaluation before administration of the first cycle), the data obtained from the sample serve as baseline values. All clinical and biological wastes from the studies were disposed according to the Veterinary Oncology guidelines with the use of anticancer agents as previously described by Théon et al. [30].

### 2.5. Physical and Clinical Observation

Daily physical, clinical signs against toxicity and mortality were observed. The body weight, rectal temperature, and heart rate were all measured as baseline and monitored twice weekly within the period of the study.

### 2.6. Sample Collection for Haematological Profile, Serum Biochemistry, and Cardiac Injury Biomarker Assay

Blood samples were collected in heparinized EDTA and plain tube for haematological profiling, serum function enzymes, and cardiac injury biomarkers, after 2 weeks of acclimatization period. Serial blood samples were collected once before each cycle. The samples were immediately analysed for complete blood count using Horiba Medical Scil Vet ABC Plus analyzer (Scil Vet. USA) with the whole blood centrifuged (Centrifuge Eppendorf 5424R, Germany) for 15 minutes at 4000 rpm, and the serum was stored at −80°C for analysis. Function enzymes were analysed using Dimension Xpand Plus Integrated Chemistry System (Dimension® EXL™ 200 Integrated Chemistry System, Siemens, Germany) to evaluate the level of alanine transaminase (ALT), aspartate aminotransferase (AST), creatinine (Crea), blood urea nitrogen (BUN), creatinine kinase (CK), and lactate dehydrogenase (LDH) all according to the protocol described by Chang et al. [31].

### 2.7. Cardiotoxicity Assessment Using Serum Biomarker

Canine cardiac troponin I (cTn-I) and canine NT-pro-brain natriuretic peptide (Canine NT-proBNP) were used as major biomarkers for cardiac injury evaluation due to their selectivity and specificity to cardiac injury according to Cartwright et al. [32] and Sawaya et al. [33]. A sandwich ELISA method was adopted using Canine cTn-I/TNNI3 (canine troponin I) and Canine NT-proBNP (N-Terminal Pro-Brain Natriuretic Peptide) ELISA kit (E-EL-C0210) specific for serum samples collected from the dogs which were processed according to manufacturer's instructions and recommendations (Elabscience Biotechnology, Co., Ltd. China). The optical densities of each well on the microplate were read at 450 nm wavelength using spectrophotometer (Tecan Infinite 200 Pro microplate reader, USA).

### 2.8. Thoracic Radiographic Evaluation

An orthogonal view of the thoracic radiograph was exposed to radiographic system during inspiration as previously suggested by Gülanber et al. [34]. DRX-1 mobile radiography (Econet Orange 10040HF model Plus Carestream image system 3543C, USA) was used to evaluate the heart size using the vertebral heart score (VHS) as previously described by Surachetpong and Teewasutrakul [35]. These procedures were performed once at the beginning of the study and once at the end of the 3 weeks after the 5th dose, with the both the long and short axes expressed in centimetres.

### 2.9. Electrical Conductivity and Cardiac Function Assessment Using Electrocardiography (ECG)

Electrocardiograms were recorded in all the dogs before the commencement of the experiment to rule out cardiac dysfunction and cardiac chamber enlargements and were repeated at the end of the three weeks after the 5th dose using the electrocardiographic system (Cardiofax GEM-9020K, Nihon, Japan) according to the protocol described by Alves de Souza and Camacho [36].

The electrodes were placed on the dogs in the standing position with crocodile clips placed on the skin just below the elbow joints and little above the stifle joint area with conducting medium added to enhance contact. The electrical activities of the heart were evaluated for the duration of 1 minute, with the paper speed of the ECG at 50 mm/sec, a voltage calibration scale of 1 cm equivalent to 1 mV, and 50 Hz filter, with the dogs manually restrained with insulated hands using gloves in the standing position devoid of panting, muscular tremor, and shaking, and at least 3 QRS complexes were recorded on the traces.

Heart rate, waves, intervals, duration of P wave, PR interval, QRS complex, and QT interval, and P wave amplitudes were recorded. However, 20% increase or decrease from any of the ECG parameters as compared to the pretreatment value obtained was considered alteration due to the intervention as previously reported by Mauldin et al. [37].

### 2.10. Euthanasia, Postmortem Examination, and Morphometry

The dogs were euthanized 3 weeks after the 5th last dose. Carcasses were subjected to postmortem examination. The heart and kidney were removed with the relative heart weight measured. An incision was made between the papillary muscles and chordal tendon to expose both left and right ventricle walls and the interventricular septum. The ventricle wall thickness was measured in millimetre using a vernier caliper with the relative heart weight calculated by a formula adopted from Fracasso et al. [38]:

(1) $$\begin{array}{c} \text{Relative organ weight }\left(\text{g}/\text{kg}\right)=\left(\text{heart weight }\left(\text{g}\right)/\text{body weight }\left(\text{kg}\right)\right)\;*\;100. \end{array}$$

### 2.11. Histopathology Analysis

The heart (left and right ventricular walls and interventricular septum) and kidney tissues were fixed in 10% buffered formalin and processed for histological evaluation as described by Schäfer-Somi et al. [39]. The blocks were sectioned to approximately 4 *µ*m in size with a microtome (Leica 2235 Microtome, USA) and then finally, stained with Harris's haematoxylin and eosin and Masson's trichrome for histochemistry and examined under the light microscope (Leica DM4M, NY USA), with Moticam Pro 282A 5.0MP (Motic images Software Plus 2.0 TWAIN, Hong Kong). The degree of tissue injury, necrosis, and inflammatory response were evaluated using qualitative and semiquantitative grading system by a pathologist blinded to the study group, as previously described by Erboga et al. [40].

### 2.12. Ultrastructural Studies

The heart and kidney tissues were selected for ultrastructural studies. The tissues were trimmed to 2–4 mm slice and fixed in 2.5% glutaraldehyde containing 0.2 M sodium cacodylate buffer (pH 7.2) for 12 hours at 4°C and were further processed in accordance with the rib method described by Bakar et al. [41]. The embedded block was trimmed to form a trapezoidal shape. An ultrathin section (50–70 nm thick) was cut using a diamond knife, which was mounted on a copper grid and then stained with uranyl acetate and lead citrate. The tissues were viewed and examined under the high-resolution transmission electron microscope (HRTEM, JEOL, JEM2100F, USA) at 80 kV with the operator blinded to the group, and 5 to 8 micrographs were taken randomly.

### 2.13. Statistical Analysis

All the data were analysed using GraphPad Prism version 7 (Prism GraphPad Software, Inc., USA). Descriptive statistics and graphs were used for the clinical signs and observations. However, sample size in all the groups is equal, the results were expressed as mean and standard deviation, and one-way ANOVA (analysis of variance) with Turkey's multiple range test method was used for multiple comparisons for haematological parameters and serum biochemical value while Kruskal–Wallis test was used for cardiac biomarker and histological tissue scoring, with a *p* value < 0.05 considered statistically significant in all the analysis.

## 3. Results

### 3.1. Physical and Clinical Observation and Mortality

**Figure 1:**
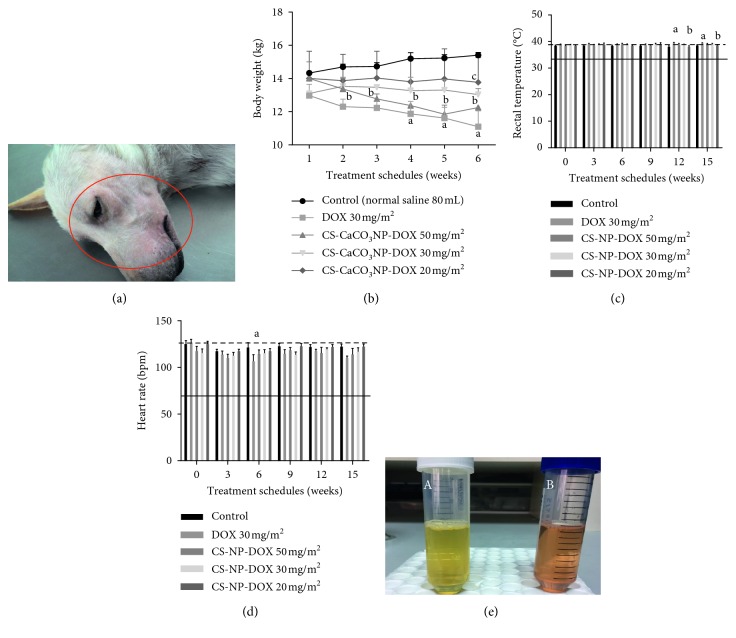
Effect on physical and clinical parameters. (a) Dermal lesion on the facial region of the dogs given free DOX after the 4th cycle. (b) Effect of treatment groups from onset to 3 weeks after the 5th cycle on the body weight (kg). (c) Effect of treatment groups from onset to 3 weeks after the 5th cycle on rectal temperature (°C). (d) Effect of treatment groups from onset to 3 weeks after the 5th cycle on heart rate (bpm). Values are expressed as mean ± standard deviation. Different alphabets indicate statistical significance (*p* < 0.05) between groups at different cumulative doses with a, b, c, and d representing significant difference with control, DOX 30 mg/m^2^, CS-CaCO_3_NP-DOX 50 mg/m^2^, CS-CaCO_3_NP-DOX 30 mg/m^2^, and CS-CaCO_3_NP-DOX 20 mg/m^2^, respectively. (e) Urine colour change in dogs given free DOX and CS-CaCO_3_NP-DOX at different doses.

No mortality and evidence of cardiotoxicity were observed in all the dogs within the study period. However, anorexia, soft watery faeces, and dermal lesions, which were expressed as alopecia with pruritus on the facial region, were observed after the 4th cycle (cumulative dose of 120 mg/m^2^) of free DOX in all 2 out of 3 dogs as a predetermined toxicity sign as shown in Figure 1.

#### 3.1.1. Effect of Free DOX and CS-CaCO_3_NP-DOX at Different Cumulative Doses on Body Weight

A decrease in body weight of dogs given 30 mg/m^2^ free DOX was observed immediately after the 2nd dose with about 9% of the body weight lost after a cumulative dose of 150 mg/m^2^. Similarly, a decrease in body weight (8.3%) was also observed in dogs given CS-CaCO_3_NP-DOX (50 mg/m^2^) at a cumulative dose of 200 mg/m^2^ as compared to the initial body weight.

However, an increase in body weight was observed in dogs given CS-CaCO_3_NP-DOX at 30 mg/m^2^ and 20 mg/m^2^ 3 weeks after the second dose as shown in Figure 1. The decrease in body weight observed in dogs given a cumulative dose of free DOX (150 mg/m^2^) and cumulative dose of CS-CaCO_3_NP-DOX (250 mg/m^2^) was significant as compared to those given normal saline (*p*=0.02 and *p*=0.014), respectively, within the last 3 weeks of the study. However, the increase in body weight observed was also significant in dogs given cumulative dose of CS-CaCO_3_NP-DOX (100 mg/m^2^) and below when compared to those given free DOX (*p*=0.008) within the last 6 weeks of the experiment.

#### 3.1.2. Effect of Free DOX and CS-CaCO_3_NP-DOX at Different Cumulative Doses on Rectal Temperature

A slight increase in the rectal temperature ranging from 0.5 to 1°C was observed in all the dogs within 12 hours after administration. However, the increase in the rectal temperature upon administration of cumulative dose of free DOX 120 mg/m^2^ as compared to that of normal saline and cumulative dose of CS-CaCO_3_NP-DOX (dose range between 120 and 150 mg/m^2^) was statistically significant with *p* ≤ 0.0001, *p* ≤ 0.0001, and *p*=0.027, respectively, after the 3 drug administrations (Figure 1).

#### 3.1.3. Effects of Free DOX and CS-CaCO_3_NP-DOX at Different Cumulative Doses on Heart Rate

No significant change in the heart rates was observed throughout the 15 weeks of the free DOX and CS-CaCO_3_NP-DOX administration at different doses, with normal heart sound originating from the sinus node except for the dogs given free DOX at a cumulative dose of above 120 mg/m^2^ that exhibited shallow heart sound. There was also a significant decrease in the heart rate in the dogs given cumulative dose of above 90 mg/m^2^ as compared to the dogs given normal saline (*p*=0.017) as shown in Figure 1.

### 3.2. Urinalysis

No significant changes in the urine samples of the dogs given CS-CaCO_3_NP-DOX at different doses were observed as compared to those given normal saline (Figure 1). However, slight change in urine colour was observed from transparent-yellowish colour to amber colour 8 hours after the 3rd cycle of free DOX administration.

### 3.3. Thoracic Radiological Findings and Mean Vertebral Heart Size

**Figure 2:**
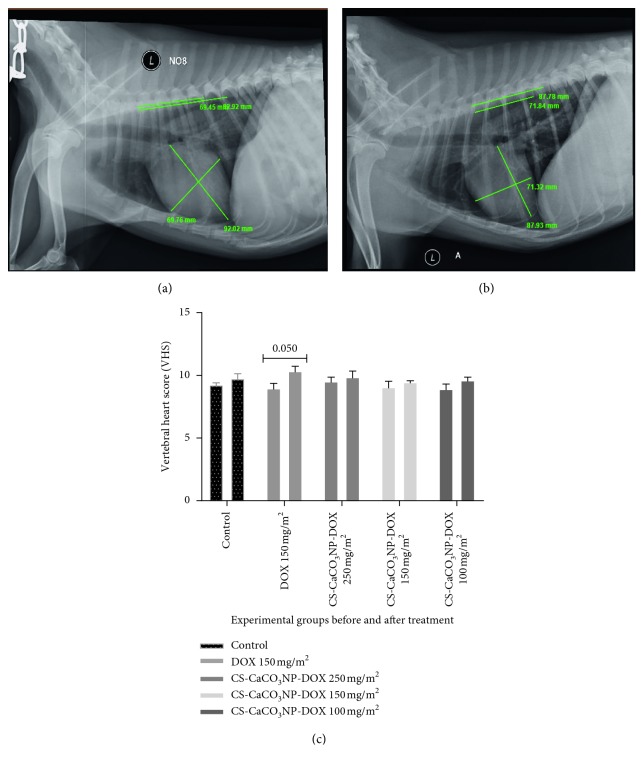
Thoracic radiographs and heart size score. (a) Initial and (b) final heart size measurement using vertebral heart score (VHS) index of dogs. (c) Mean vertebral heart score of dogs treated with free DOX and CS-CaCO_3_NP-DOX at different cumulative doses. All the data are expressed as mean ± SD, *n*=3.

The right lateral radiographic image of the dogs given free DOX 30 mg/m^2^ at a cumulative dose of 150 mg/m^2^ revealed a large cardiac silhouette on the ventricular border with cranial bulge close to the sternum. The cardiac apex was slightly displaced with the conus arteriosus on the dorsoventral view (Figure 2).

A mean increase in vertebral heart score (VHS) of 1.36 ± 0.55 v was observed in the dogs given free DOX at a cumulative dose of 150 mg/m^2^ as compared to the baseline VHS value. The increase in VHS was not statistically significant in the groups of dogs given CS-CaCO_3_NP-DOX at different cumulative doses as compared to the respective baseline VHS value (*p*=0.0501). Similarly, the *p* values for the cumulative doses in dogs given CS-CaCO_3_NP-DOX (250 mg/m^2^), CS-CaCO_3_NP-DOX (150 mg/m^2^), and CS-CaCO_3_NP-DOX (100 mg/m^2^) are 0.2354, 0.3394, and 0.1088, respectively, when compared to their respective baseline values (Figure 2).

### 3.4. Electrocardiographic Findings with Cumulative Doses of Free DOX and CS-CaCO_3_NP-DOX

**Figure 3:**
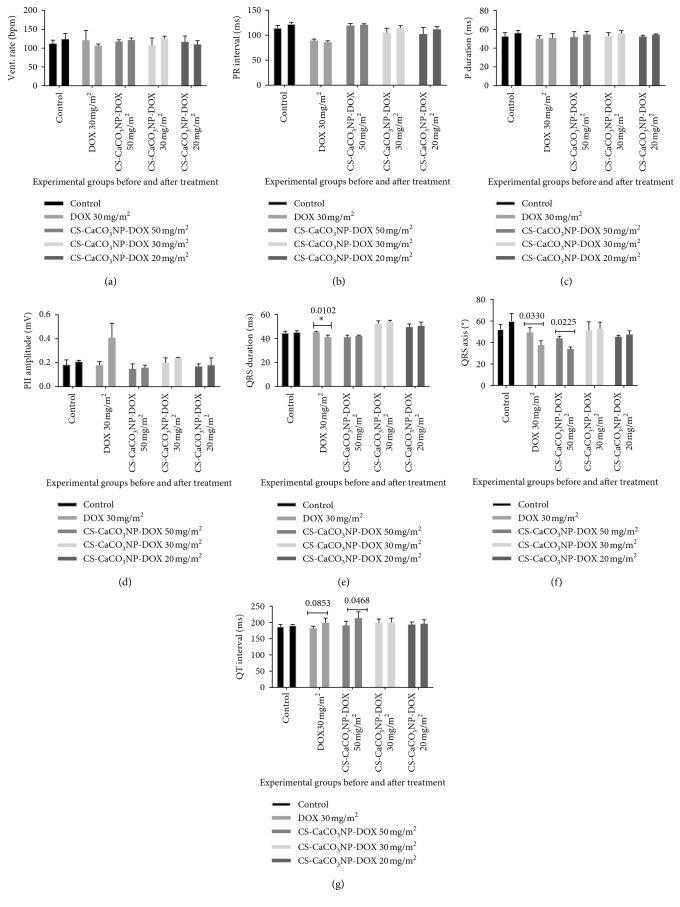
Electrocardiogram changes due to cumulative free DOX 150 mg/m^2^ and different CS-CaCO_3_NP-DOX cumulative formulation regimen ranging from 100 to 250 mg/m^2^. (a) Ventricular rate, (b) PR interval, (c) P wave, (d) PII amplitude, (e) QRS duration, (f) QRS axis, and (g) QT interval. Values are expressed as mean ± standard deviation. *p* < 0.05 is considered significant.

No change was observed in the ventricular rate and PR interval in dogs when compared with the baseline values before administration of the drug (Figure 3), respectively. The ECG recorded a significant decrease in the duration of P wave in dogs given a cumulative dose of free DOX 150 mg/m^2^ as compared to the baseline P wave (*p*=0.0125) (Figure 3). In addition, the changes in the P wave were within the normal reference range in the dogs given CS-CaCO_3_NP-DOX and the changes in the PII amplitude were also observed, although there was an increase in the PII amplitude in the dogs given free DOX (Figure 8). However, a significant decrease in QRS duration was detected in the dogs given free DOX at a cumulative dose (150 mg/m^2^) as compared to the initial baseline data (*p*=0.0134) (Figure 3). In addition, the ECG also reveals a significant decrease in QRS axis in the dogs given cumulative doses of free DOX (150 mg/m^2^) and CS-CaCO_3_NP-DOX (250 mg/m^2^) with *p*=0.0166 and *p*=0.0188, respectively (Figure 3).

However, there is a mean significant increase in the QT interval in the dogs given free DOX 3 weeks after a cumulative dose of 150 mg/m^2^ (*p*=0.0357) and in those given CS-CaCO_3_NP-DOX 250 mg/m^2^ (*p*=0.0464) as compared to the baseline values (Figure 3).

### 3.5. Haematology, Serum Biochemistry, and Biomarkers for Cardiac Injury

#### 3.5.1. Effect of Free DOX and CS-CaCO_3_NP-DOX at Different Cumulative Doses on Haematological Profile

**Figure 4:**
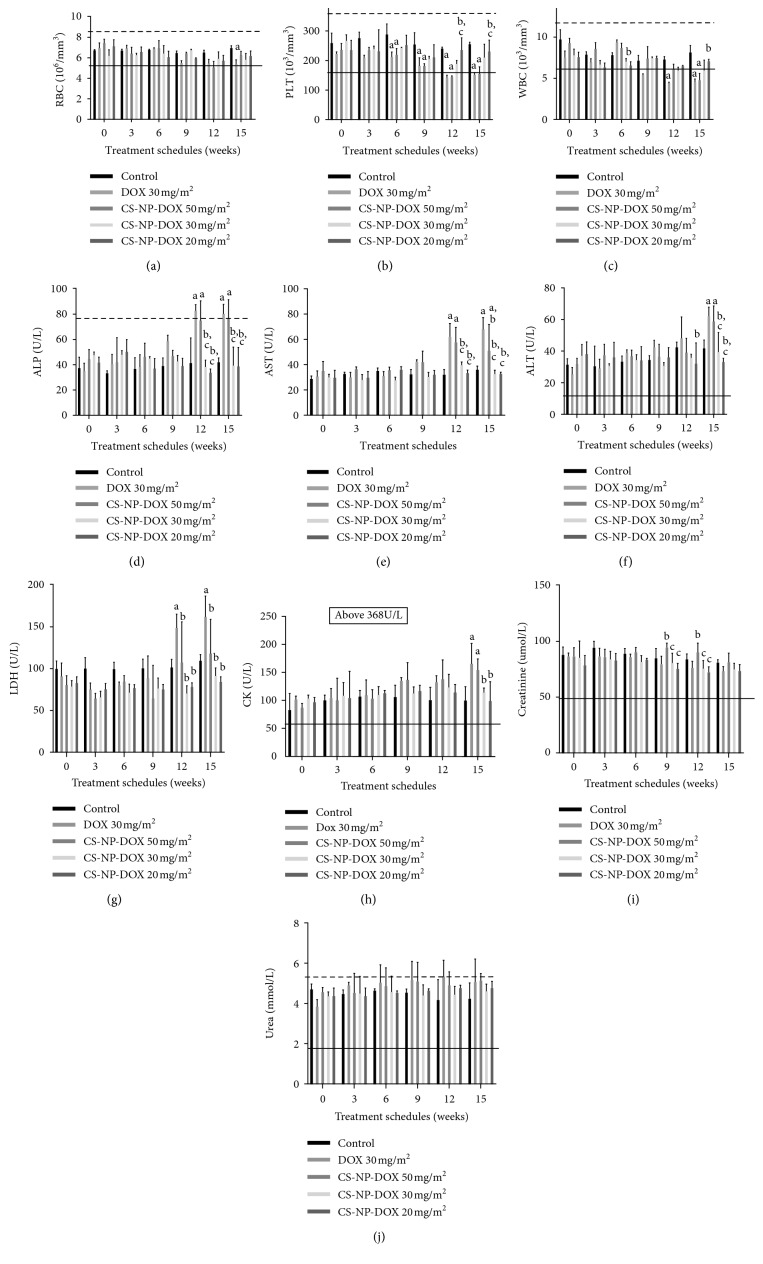
Effect of treatment groups from onset to 3 weeks after the 5th cycle on the haematological profile and serum biochemistry. (a) Red blood cells (RBCs) (10^6^/mm^3^), (b) platelet cells (PLT) (10^3^/mm^3^), (c) white blood cells (WBC) (10^3^/mm^3^), (d) alkaline phosphatase (ALP) (*µ*/L), (e) aspartate aminotransferase (AST) (*µ*/L), (f) alanine aminotransferase (ALT) (*µ*/L), (g) lactate dehydrogenase (LDH) (*µ*/L), (h) creatinine kinase (CK) (*µ*/L), (i) creatinine (Crea) (*µ*mol/L), and (j) urea (mmol/L). Values are expressed as mean ± standard deviation. Different alphabets indicate statistical significance (*p* < 0.05) between groups at different cumulative doses with a, b, c, and d representing significant difference with control, DOX 30 mg/m^2^, CS-CaCO_3_NP-DOX 50 mg/m^2^, CS-CaCO_3_NP-DOX 30 mg/m^2^, and CS-CaCO_3_NP-DOX 20 mg/m^2^, respectively.

Haematological changes observed were dose-related specifically on the red blood cell (RBCs), platelet cells (PLTs), and white blood cells (WBCs) with significant changes at a different level when compared to the respective cell levels in the dogs given normal saline. Significant variations were observed in the dogs given cumulative dose of above 90 mg/m^2^ and CS-CaCO_3_NP-DOX 150 mg/m^2^ and above as compared to the cell levels in the dogs given normal saline. As seen with RBC (*p*=0.0079), after a cumulative dose of free DOX 150 mg/m^2^, and PLT (<0.05), at cumulative dose of 60 mg/m^2^, there is a mean significant decrease in the WBC at a cumulative dose of 90 mg/m^2^ as compared to the dogs given normal saline (*p*=0.007) (Figure 4). However, no significant alteration in the cell profiles in the dogs given CS-CaCO_3_NP-DOX (30 mg/m^2^) at a cumulative dose of 150 mg/m^2^ and below was observed as compared to the dogs given normal saline.

#### 3.5.2. Effect of Free DOX and CS-CaCO_3_NP-DOX at Different Cumulative Doses on Serum Enzyme Function Level

The liver, renal, and structural membrane injury biomarkers were monitored, with no significant increase in the mean ALP value in the dogs given CS-CaCO_3_NP-DOX 150 mg/m^2^ and below as compared to the baseline value and to the mean serum level in those given normal saline (*p*=0.9964 and *p*=0.9914) (Figure 4). However, a significant change in the mean level was observed in the dogs given free DOX at a cumulative dose of 120 mg/m^2^ and CS-CaCO_3_NP-DOX at dose above 150 mg/m^2^ when compared to the baseline value and mean levels in those given normal saline *p* ≤ 0.0001 and *p*=0.0003, respectively (Figure 4). In addition, similar pattern of mean serum elevation in AST was observed with *p* ≤ 0.0001 and *p* ≤ 0.0001 in dogs given free DOX 90 mg/m^2^ and CS-CaCO_3_NP-DOX at cumulative dose of above 200 mg/m^2^, respectively (Figure 4), whereas the mean ALT level was significantly higher with free DOX and CS-CaCO_3_NP-DOX at cumulative dose of above 150 mg/m^2^ when compared to the mean level in the dogs given normal saline *p*=0.0052  and  0.0285, respectively (Figure 4); however, LDH, CK, Crea, and BUN all show similar pattern of the elevation in the enzyme level as compared to the baseline mean value and the corresponding levels in the dogs given normal saline (Figure 4).

#### 3.5.3. Quantification of Serum Canine Cardiac Troponin I (cTn-I)

**Figure 5:**
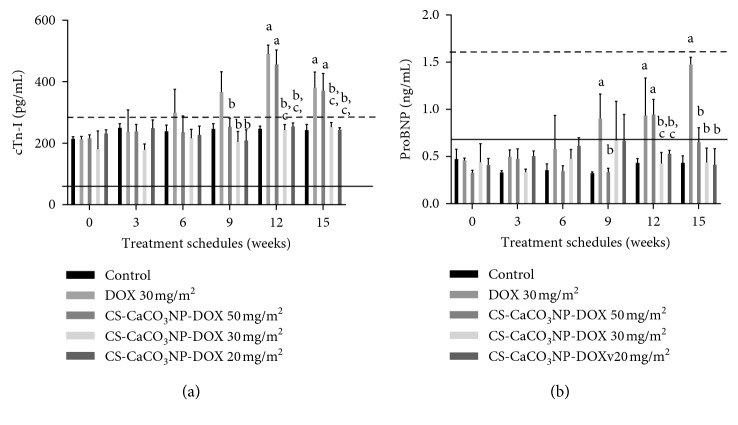
Cardiac injury biomarker. (a) Mean cardiac troponin I (cTn-I) concentration (pg/ml). (b) Mean N-terminal pro-brain natriuretic peptide (proBNP) concentration (ng/ml) of treatment groups from onset to 3 weeks after the 5th dose. Values are expressed as mean ± standard deviation. Different alphabets indicate statistical significance (*p* < 0.05) between groups at different cumulative doses with a, b, c, and d representing significant difference with control, DOX 30 mg/m^2^, CS-CaCO_3_NP-DOX 50 mg/m^2^, CS-CaCO_3_NP-DOX 30 mg/m^2^, and CS-CaCO_3_NP-DOX 20 mg/m^2^, respectively.

The serum cardiac troponin cTn-I concentration of the dogs over the period of the study is shown in Figure 5. The serum cTn-I was significantly higher in the dogs given free DOX (30 mg/m^2^), after cumulative dose (90 mg/m^2^) (*p*=0.0003), and at the end of the experiment 15 weeks cumulative dose of 150 mg/m^2^ (*p*=0.0045) as compared to serum cTn-I of the control dogs. There was also an increase in the serum biomarker in dogs given CS-CaCO_3_NP-DOX 50 mg/m^2^ (150 mg/m^2^) (*p*=0.0083) and at cumulative dose of 250 mg/m^2^ (*p*=0.0091) as compared to those in the control group. In addition, the mean levels of serum cardiac troponin in dogs given free DOX at a cumulative dose of above 90 mg/m^2^ were significantly higher as compared to those given cumulative doses of the CS-CaCO_3_NP-DOX 150 mg/m^2^ and below (Figure 5).

#### 3.5.4. Quantification of Serum NT-Protein Brain Natriuretic Peptides (proBNP)

A significant increase in the proBNP was recorded after a cumulative dose of 60 mg/m^2^ in dogs given free DOX 30 mg/m^2^ as compared to the level of proBNP in the dogs in the control group (*p*=0.0042). In addition, a significant elevation in NT-proBNP in the dogs given a cumulative dose of CS-CaCO_3_NP-DOX 50 mg/m^2^ (200 mg/m^2^) was observed when compared to the mean concentration to the corresponding level of the control group. However, NT-proBNP in the serum at a cumulative dose of CS-CaCO_3_NP-DOX 50 mg/m^2^(80 mg/m^2^ and above) significantly differs from the mean level of the NT-proBNP in dogs given a cumulative dose of 60 mg/m^2^ of the free DOX (Figure 5).

### 3.6. Gross Anatomical Alternations at Necropsy

**Figure 6:**
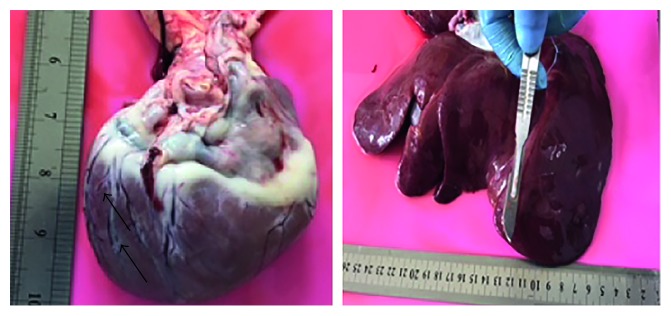
Gross pathological lesions on the heart and liver of dogs given cumulative dose of free DOX 150 mg/m^2^.

Grossly, mild pinpoint haemorrhage was observed on the left ventricular wall of the heart in 2 out of 3 dogs given cumulative dose of free DOX 150 mg/m^2^ with swallowed edges of the liver and the right lobe appeared rounded in all 3 dogs as compared to the liver of the control of dogs given normal saline. However, the kidney appeared normal with no gross lesions. In contrast, the heart and kidneys of the dogs given different doses of CS-CaCO_3_NP-DOX and normal saline did not show any gross pathological lesions as shown in Figure 6.

#### 3.6.1. Morphometry of the Body Weight, Heart Weight, and Ventricular Mass Measurement

**Figure 7:**
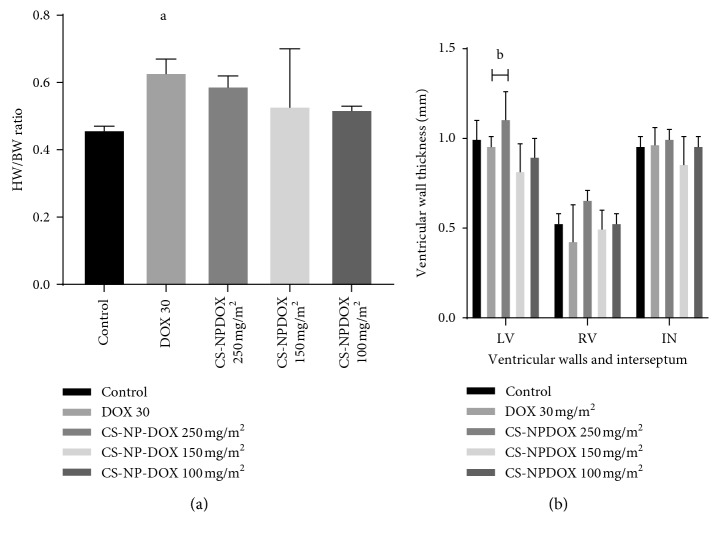
Morphometric assessment of the heart. (a) Mean heart weight-to-body weight ratio. (b) Mean ventricular wall thickness (mm) of dogs given normal saline, cumulative dose of free doxorubicin 150 mg/m^2^, CS-CaCO_3_NP-DOX 250 mg/m^2^, CS-CaCO_3_NP-DOX 150 mg/m^2^, and CS-CaCO_3_NP-DOX 100 mg/m^2^ (*n*=3). *p* < 0.05 is considered statistically significant between the groups.

The heart weight-to-body weight ratio increased significantly in dogs given free DOX at a cumulative dose of 150 mg/m^2^ as compared to those given normal saline (*p*=0.0020), whereas the dogs given different doses of CS-CaCO_3_NP-DOX did not show any significant change as compared to those dogs given normal saline (Figure 7). There was a significant decrease in the left ventricular wall thickness in the dogs given free DOX at a cumulative dose of 150 mg/m^2^ as compared to those given CS-CaCO_3_NP-DOX at a cumulative dose of 250 mg/m^2^ (*p*=0.00341). However, neither increase nor decrease was observed in the right ventricular thickness of all the dogs (Figure 7).

### 3.7. Histopathological Alteration on the Cardiac and Kidney Tissues due to Cumulative Doses of DOX and CS-CaCO_3_NP-DOX

The microscopic investigation revealed that no pathological lesions were observed on the myocardium and kidney tissues of dogs given normal saline, which was similar to those given CS-CaCO_3_NP-DOX at a cumulative dose of 150 mg/m^2^ and below. However, the significant changes were observed in the tissues of dogs given free DOX at a cumulative dose of 150 mg/m^2^ and dogs given a cumulative dose of CS-CaCO_3_NP-DOX above 150 mg/m^2^.

**Figure 8:**
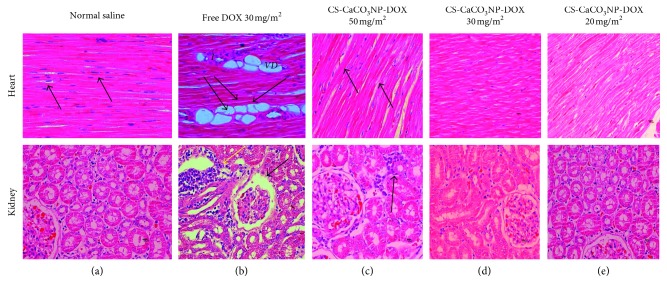
Photomicrograph of the longitudinal section of left ventricular myocardium and kidney section. (a) Ventricular myocardium and kidney section of the dogs given normal saline showing normal morphological architecture of the cardiomyocytes, myocardial fibres, and both glomerular (G) and renal tubular structure (PT). (b) Myocardium of the dogs given free DOX 150 mg/m^2^ showing myofibrillar loss, myocardial fibre disruption, multifocal cytoplasmic vacuolation (V), cardiomyocyte disorientation, cellular infiltration (I), and myocytolysis (M) with the kidney section showing dilated glomerular capsular space and dilation of the proximal renal tubular (DPT) with severe intercellular infiltration. (c) Myocardium of the dogs given CS-CaCO_3_NP-DOX 250 mg/m^2^ showing myofibrillar loss, vacuolation (v), cardiomyocyte disorientation, and cellular infiltration with the kidney section showing dilated renal tubules and focal areas of congestion. (d) Myocardium of the dogs given CS-CaCO_3_NP-DOX 150 mg/m^2^ showing normal myofibril with spindle-shaped centrally placed cardiomyocytes with the kidney section revealing normal architecture glomerulus and renal tubules. (e) Myocardium and kidney section of the dogs given CS-CaCO_3_NP-DOX 100 mg/m^2^ showing normal myofibril structure and normal structural morphology of glomerulus and renal tubules (H & E) Scale bar 10 *µ*m.

The histological sections of ventricular myocardium and kidney tissues of the dogs given normal saline and CS-CaCO_3_NP-DOX at a cumulative dose of 150 mg/m^2^ appeared normal as shown in Figure 8. The histological sections of the left ventricle of dogs given cumulative doses of 150 mg/m^2^ of free DOX showed multiple areas of myocardial intercellular vacuolation of different sizes which disrupts the myofilaments with clusters of inflammatory infiltration cells predominantly lymphocytes (Figure 8).

In addition, focal myocytolysis was also seen in some areas which appeared as myofibril derangement of myocardial fibres associated with halo vacuoles, which are area of depleted glycogen and thus replaced by collagen as compared to the histological section of left ventricular myocardial of the dogs given normal saline which retained its myocardial architecture morphology with clearly distinct dense central nucleus (Figure 8). Furthermore, there was no myocyte disruption in dog's ventricle and the appearance of cytoplasmic bridges at the end of myofilaments in dogs given either CS-CaCO_3_NP-DOX at a cumulative dose of 150 mg/m^2^ or with less concentration as shown in Figure 8.

The focal haemorrhagic area was observed in the intercellular region of myofibril in the dogs given free DOX and higher cumulative dose of CS-CaCO_3_NP-DOX above 150 mg/m^2^ (Figure 8). The myocardium of the left and right ventricles was morphologically normal with spindle-shaped central nucleus without inflammatory cell infiltration in dogs given normal saline and in dogs given CS-CaCO_3_NP-DOX at a cumulative dose of 150 mg/m^2^ or less (Figure 8).

In the kidney, focal necrosis was observed in the renal parenchyma leading to distortion of the parenchymatous region with high number of basophilic tubular cells and dark pinpoint fragmentation of the renal tubular epithelial nuclei, few areas of haemorrhages, and slight dilation of few renal proximal tubules of the dogs given cumulative of free DOX 150 mg/m^2^ (Figure 8). In addition, decrease in the renal glomerular cellularity associated with atrophied glomerulus resulting in widening of Bowman's capsular space was observed in the dogs given cumulative dose of free DOX 150 mg/m^2^ as compared to the control group, which shows normal morphological appearance of the renal corpuscle with tuft glomeruli and Bowman's capsule space and narrowed luminal proximal convoluted tubules lined with cuboidal cells as shown in Figure 8. However, a similar focal necrotic area with renal tubular nuclear fragmentation and disrupted epithelium was observed in the dogs given CS-CaCO_3_NP-DOX at a cumulative dose of 250 mg/m^2^ as compared to the control group (Figure 8). No alteration was observed in the tubular, glomerular, and parenchymatous region in the kidney of dogs given CS-CaCO_3_NP-DOX at a cumulative dose of 150 mg/m^2^ and below (Figure 8), respectively.

**Figure 9:**
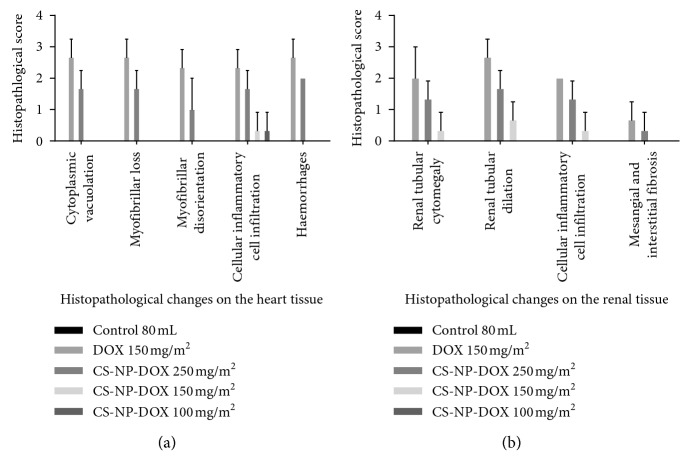
Semiquantitative analysis of both left ventricular myocardial muscle and the renal tissue of the experimental dogs. All the data are expressed as mean ± SD.

A significant difference (*p* < 0.001) was observed in the intercellular vacuolation of the left ventricles between the groups (*χ*^2^ = (4, 180) = 26.211). In addition, significant difference was observed in the histological structures of ventricular myocardium in dogs given free DOX at cumulative dose of 150 mg/m^2^ and those given CS-CaCO_3_NP-DOX at cumulative doses of 250 mg/m^2^, 150 mg/m^2^, and 100 mg/m^2^ with *p* < 0.001, *r*=0.198; *p* < 0.001, *r*=0.05; *p*=0.154, *r*=0.02; and  *p*=0.154, *r*=0.02, respectively, as compared to the control group (Figure 9).

The vacuolation in the ventricular wall observed in the experiment groups was directly related to the significant loss in myofibril (*χ*^2^ = (4, 180) = 40.138, *p* < 0.001). The vacuolation in the ventricular wall was significantly more in dogs given free DOX at cumulative dose of 150 mg/m^2^ and those given CS-CaCO_3_NP-DOX at cumulative doses of 250 mg/m^2^ with *p* < 0.001, *r*=0.259 and *p* < 0.001, *r*=0.238, respectively, as compared to those in the control group, while there was no significant difference in dogs given CS-CaCO_3_NP-DOX at cumulative dose of 150 mg/m^2^ or lower (*p*=0.079, *r*=0.043) and (*p*=1.000, *r*=0.014) when compared to those in the control group (Figure 9).

The significant difference in the cytoplasmic interstitial cellular infiltration and haemorrhages was observed among the groups (*χ*^2^ = (4, 180) = 13.068, *p*=0.011 and *χ*^2^ = (4, 180) = 18.166, *p*=0.001), respectively, which were significantly higher in dogs given free DOX at cumulative dose of 150 mg/m^2^ and those given CS-CaCO_3_NP-DOX at cumulative doses of 250 mg/m^2^, 150 mg/m^2^, and 100 mg/m^2^ with (*p*=0.003, *r*=0.124), (*p*=0.006, *r*=0.107), (*p*=0.154, *r*=0.028), and  (*p*=0.079, *r*=0.043), respectively, as compared to those given normal saline (Figure 9).

#### 3.7.1. Histochemistry Evaluation of the Effect of Cumulative Doses of DOX and CS-CaCO_3_NP-DOX on Cardiac and Hepatic Tissues

**Figure 10:**
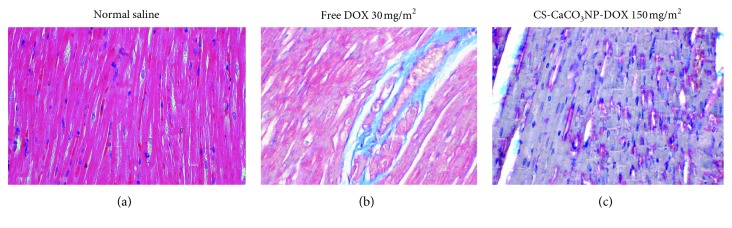
Photomicrograph of the myocardium. (a) Myocardium of the dogs given normal saline showing the normal morphological architecture of myocardial fibres. (b) Free DOX 150 mg/m^2^ showing collagen deposit within the interstitial myocardial space of myocardial fibres. (c) CS-CaCO_3_NP-DOX 150 mg/m^2^ showing basophilic cardiomyocytes with the myocardial fibres (Masson's trichrome). Scale bar 10 *µ*m.

The histological sections of the myocardium of both dogs given normal saline and CS-CaCO_3_NP-DOX at a cumulative dose of 150 mg/m^2^ and below revealed similar normal morphology. They showed normal distribution of collagen between the myofibrils (Figure 10). However, a marked deposition of collagen fibrils was observed in the interstitial myofibril space between myocardial filaments in dogs given a cumulative dose of free DOX 150 mg/m^2^ (Figure 10). Moreover, dense collagen matrix was also observed well deposited in the myomysium between the myofibrils and appeared as blue-stained materials in the myocardial tissue (Figure 10).

### 3.8. Ultrastructural Changes in Cardiac and Kidney Tissues of Dogs Given Cumulative Doses of DOX and CS-CaCO_3_NP-DOX

**Figure 11:**
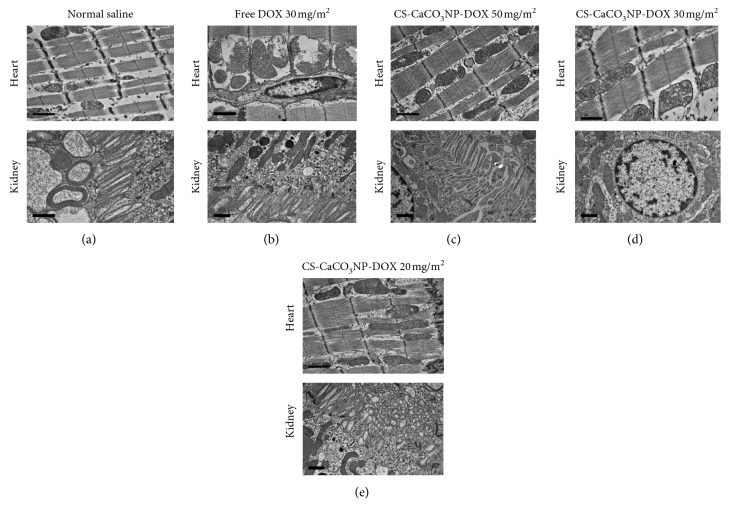
Electron micrograph (TEM) of the left ventricular myocardial tissue section and kidney section. (a) Myocardial tissue section of dogs given normal saline showing clear distinct cisternae on the mitochondria, with spindle-shaped nucleus within the myofilament and the proximal convoluted tubules of the dogs given normal saline revealing its lumen and apical cellular membrane with microvilli appearing normal with distinct mitochondrial cisternae and nerves surrounded by myelin sheaths. (b) Free DOX 150 mg/m^2^ showing extensive mitochondrial damage and rupture with marked swelling and cristae disorientation, scattered vacuolated cardiomyocyte, with free empty vacuum and *Z*-band aggregate in close cluster to ribosome in the cytoplasm with the dilated proximal convoluted tubules with vesicle and distorted cristae in the mitochondria. (c) CS-CaCO_3_NP-DOX 250 mg/m^2^ showing mitochondrial rupture with cristae disorientation with free empty vacuum with few dilated proximal convoluted tubules and shrinked apical microvilli and vesicle and sarcoplasmic tubules. (d) CS-CaCO_3_NP-DOX 150 mg/m^2^ showing clear distinct cisternae on the mitochondria, with sarcoplasmic tubules, myofilament with the renal nucleus with distinct peripheral distribution of chromatic material and normal appearance with mild swollen mitochondrial and rough endoplasmic reticulum with a normal basement membrane thickness. (e) CS-CaCO_3_NP-DOX 100 mg/m^2^ showing clear distinct cisternae on the mitochondria, with sarcoplasmic tubules, myofilament with the normal appearance of renal tissue with mild swollen mitochondrial and rough endoplasmic reticulum with a normal basement membrane thickness (1 *µ*m).

The electron micrograph of the myocardium of dogs given free DOX at a cumulative dose of 150 mg/m^2^ revealed extensive mitochondrial rupture with marked swelling and cristae disorientation (Figure 11). The orientations of the myofibrils were irregular with vacuolation within the intercellular space of the myofibrils with clusters of electron dense deposit within the sarcomere and intermyofibrillar cytoplasm as shown in Figure 11. In addition, scattered vacuolated cardiomyocyte with free empty vacuum, focal disruption of myofilament, and Z-band aggregate in close cluster to the ribosome in the cytoplasm are indicatives of myofibril loss in the left ventricular electron micrograph of the dogs given free DOX at a cumulative dose of 150 mg/m^2^ (Figure 11). Similar changes were observed on the TEM of the myocardium in dogs given a higher dose of CS-CaCO_3_NP-DOX (at a cumulative dose of 250 mg/m^2^), and marked mitochondrial swelling was observed as compared to the ruptured mitochondria in dogs given free DOX at a cumulative dose of 150 mg/m^2^ (Figure 11), whereas a clear regular orientation of the myofibrils with distinct cisternae on the mitochondria with oval- to spindle-shaped nucleus was observed in the myocardial tissue in dogs given normal saline and those given CS-CaCO_3_NP-DOX at a cumulative dose of 150 mg/m^2^ and below (Figure 11).

The TEM of renal tissue in the dogs given normal saline revealed normal architectural morphology of the apical region of the proximal convoluted tubules with microvilli membrane associated with distinct mitochondrial cisternae and nerves enveloped by myelin sheaths (Figure 11), which were similar to those given CS-CaCO_3_NP-DOX at cumulative dose of 150 mg/m^2^ and below. Furthermore, the renal nucleus was surrounded by the peripheral distribution of chromatin material in close relation with normal mitochondria, rough endoplasmic reticulum, and basement membrane (Figure 11). However, those given free DOX 150 mg/m^2^ and CS-CaCO_3_NP-DOX at a cumulative dose of above 150 mg/m^2^ have dilated proximal convoluted tubules, distorted mitochondrial cisternae, vesicle within the renal cytoplasmic region, and the presence of numerous lysosomes and peroxisomes (Figure 11).

## 4. Discussion

Currently, several innovative developments are witnessed within nanomedicine, specifically in the area of applied medicine [42]. In spite of all the advances in chemotherapeutic formulation development, toxicity of nano-DOX formulation still remains as a major setback, since most of the organic nanocarrier used themselves causes tissue damage [42], leaving oncologist with no other option but rather to amend dose of DOX or combine it with another cytotoxic drug which might not be enough to inhibit the tumour metastasis and growth [1].

The primary goal of this study was to evaluate the safety and toxicity of CS-CaCO_3_NP-DOX in healthy dogs, which can only be achieved by evaluating tissue injury biomarkers and cytological examination of tissue with more emphasis on the myocardium and kidney tissues. The findings from this study, which is lacking in the scientific literature, will serve as a guide in dose selection, understanding the safety margin of the formulation for clinical application.

Alopecia is a common manifestation of the toxic effect of repeated dose administration of DOX in both clinical and experimental studies [43]. However, the encapsulation of DOX into CS-CaCO_3_NP prevents uncontrolled release and the manifestation of alopecia in the dogs. The hair loss observed in the dogs given free DOX was in agreement with what was reported by Todorova et al. [44] in dogs given repeated doses of 20–30 mg/m^2^ at the 20th day in the management of the canine mammary tumour.

Generally, a decrease in body weight is an indicator of cellular disturbance and biomarker for safety in toxicity studies as reported by Patel et al. [45] and Chen et al. [46]. It is well known that chemotherapy causes general body weight loss [43]. The body weight loss observed in the dogs given a cumulative dose of free DOX agrees with the report of Boussada et al. [43] in Wister rats and was in line with findings of Sato et al. [47] in mice and DeFrancesco et al. [48] in dogs. In this study, the weight loss is likely to be associated with anorexia, homeostatic body function, and metabolic imbalance of the electrolytes, as seen earlier in rodent given DOX [49].

However, less body weight loss observed with CS-CaCO_3_NP-DOX at a cumulative dose of 150 mg/m^2^ could be attributed to the reduction in bioavailability of DOX as a result of sustained control release of DOX from CS-CaCO_3_NP in the body. This eventually helps in reducing the cytotoxic effect associated with free DOX and further explains the safety nature of CS-CaCO_3_NP-DOX at a cumulative dose of 150 mg/m^2^ as compared to free DOX at an equivalent dose.

The mild increase in rectal temperature recorded was similar to the earlier reports of Sen et al. [50], where body temperature increased within 5th–9th hours of administration of anticancer in mice. This transient elevation could be associated with the immunological reaction between DOX and the immune cell.

The changes in the blood cells especially erythrocytes, platelets, and leucocyte count were all DOX dose dependent and were consistent with the findings reported by Manno et al. [5]; Tacar et al. [51], which could be attributed to the known bone marrow suppressive effect of DOX and iron-DOX interaction, thus causing RBC membrane rupture as previously reported by Vici et al. [52] and Pahouja et al. [53] since DOX did not limit its effects not only to cancer cell alone but also to proliferating haematopoietic cells.

The reduction in leucocytes observed in dogs given free DOX was in agreement with Judson et al. [54], who reported neutropenia and thrombocytopenia with DOX administration. In addition, a similar reduction was observed in RBC and WBC in DOX-induced toxicity in the mouse [4], cat [55, 56], and dogs [57]. It is important to note that direct platelet lysis contributes to the thrombocytopenia as seen earlier with amoxicillin and vincristine which induced platelets lysis and were reported to cause thrombocytopenia [58, 59]. Interestingly, CS-CaCO_3_NP-DOX, when given at dose regimen of 30 mg/m^2^, did not alter the haematological cells as compared to its equivalent doses of free DOX 30 mg/m^2^. However, when CS-CaCO_3_NP-DOX 50 mg/m^2^ is given, it expected that the concentration maximum could be higher after the third dose in circulation which could have resulted in the effect observed.

The serum biochemical evaluation profile provides supportive evidence for the toxicity and safety of CS-CaCO_3_NP-DOX as compared to the free DOX. The slow release of the DOX from the CS-CaCO_3_NP might have prevented excessive interaction of the DOX with normal proliferative cells leading to cellular damage due to oxidative reactions [40]. DOX causes disruption of the myocyte membrane, which triggers the release of CK and AST into the peripheral circulation [60], and thus serves as an indicator for muscular tissue injuries, associated with ALT enzymes in the liver which explains the integrity cell membrane [60].

The increase in ALP and AST in the dogs, upon cumulative dose of 120 mg/m^2^ and above of the free DOX, concords with the reports of Chen et al. [60] and is similar to the findings of Xin et al. [61], in dogs, with AST and CK increase attributed to myocardial disruption due to free oxygen radical release from DOX metabolic pathway.

The increase in AST and ALP with the cumulative dose of free DOX and a higher concentration of CS-CaCO_3_NP-DOX above 120 mg/m^2^ indicate cellular damage to the hepatic cells causes an outflow of the enzymes into the circulation which absolutely correlates with the histological tissue finding observed on the liver.

Swamy et al. [62] reported an increase in LDH and CK in the early stage of myocardial injury induced by DOX in the mouse. This report was consistent with our finding in LDH and CK levels in dogs given free DOX, although the increase in LDH was not specific for myocardial injury; however, increase in CK at cumulative dose of free DOX above 120 mg/m^2^ was observed as compared to its level in the dogs given equivalent cumulative dose of CS-CaCO_3_NP-DOX, which is suggestive of myocardial injury and renal damage. The alteration in the creatinine and urea in the dogs given cumulative dose of free DOX 150 mg/m^2^ and higher cumulative dose of CS-CaCO_3_NP-DOX 250 mg/m^2^ was similar to what was observed by Han et al. [63] in beagle dogs given 4 weeks repeated dose of camptothecin, which has similar mechanism action with DOX in terms of topoisomerase I enzyme suppression.

cTn-I is currently applied as a specific biomarker for cardiac injury in both humans and animals [64–66]; thus, it is used to access cardiac injury in mini pig [65, 67] and rats [65, 68]. However, the increase in cTn-I level was used to further confirm the histopathological changes on the myocardium. Interestingly, the pattern in which cTn-I level increases was similar to what was observed in the mice given free repeated dose of the DOX 1 mg/kg with a significant increase in the cTn level in the serum after 42 days with the level reaching a plateau level at 70 days [69].

In addition, NT-proBNP is also complimentary diagnostic assay for the monitoring and evaluating cardiac injury regardless of whether it is congestive heart failure or not [70, 71], although less specific to cTn-I. In a similar works, Wu et al. [70] reported an increase in the BNP serum concentration in rat in both short- and long-term repeated doses of DOX toxicity studies. However, vertebral heart score was used to evaluate heart size within the thoracic cavity. The mean increase in the heart size of the dogs given free DOX at a cumulative dose of 150 mg/m^2^ as compared to the dogs given CS-CaCO_3_NP-DOX agrees with the findings of Nakayama et al. [72] in the dogs, where VHS was used to assess heart size. However, cardiac silhouettes observed increases the opacity for cardiac radiograph which concurs with the findings by Guglielmini et al. [73], where fluid increases the opacity and changes the size and shape of cardiac silhouettes in the dogs with congestive heart failure.

Electrocardiography presently serves as a monitoring tool for cardiac safety assessment; however, several clinical cardiac safety assessments applied this tool to detect abnormal conductivity of the heart [7]. In this study, significant changes were observed, showing clear indication of the supraventricular arrhythmia and systolic dysfunction with ventricular enlargement which are suggestive of cardiomyopathy. The changes in QRS complex, QRS axis, and QT interval in dogs given cumulative dose of free DOX 150 mg/m^2^ were consistent with the changes in DOX-induced myocardial injury reported by Kulkarni and Swamy [7] and were in agreement with reports of Wu et al. [70] in rats and Pereira Neto et al. [74] in healthy dogs. These were all signs of either supraventricular or ventricular arrhythmias, thus a manifestation of cardiomyopathy, which could be attributed to the myofibril disruption and myofilament disorientation impulse blockage on the heart. However, encapsulation CS-CaCO_3_NP-DOX with a slow release of DOX could have prevented myocardial injury.

The supraventricular arrhythmia observed was dose-related, which could be due to multiple actions of DOX on cardiac electrophysiological activities; however, these findings were similar to the reports of Pereira Neto et al. [74] in dogs with signs of myocardial dysfunctions at a cumulative dose of DOX above 150 mg/m^2^.

The increase in the heart weight-to-body weight ratio in dogs given free DOX might be attributed to decrease in the body weight. This finding was consistent with the report of Swamy et al. [62]. Thus, the enlargement of the sarcoplasmic reticulum and mitochondrial swelling could be attributed to the increase in the heart weight-to-body weight ratio as the structural changes are often observed in DOX-induced cardiomyopathy [7].

The left ventricular wall thickness agrees with the reports of Nakayama et al. [72] that ventricular wall thinning is associated with dilated cardiomyopathy. In addition, the changes in the myocardium conform with the findings reported by Noda et al. [75] and Working et al. [76] that DOX-induced cardiotoxicity possesses unique features different from other cardiac injuries such as myocardial vacuolation, oedema, and myofibrillar membrane damage associated with interstitial fibrosis. These histopathological changes could be as a result of the free radical release from iron-dependent interaction and deregulation of the calcium channel pump with the myocytes leading to uncontrol reflux within and outside the cells.

The normal histological structure of the myocardial tissue in dogs given CS-CaCO_3_NP-DOX at cumulative dose of 150 mg/m^2^ and below further provides evidence that no cardiotoxicity was induced on the myocardial tissue in the dogs which were similar to the finding observed by Rahman et al. [77] in rabbit given DOX encapsulated in liposomal polymeric nanocarrier. The myocardial disorientation observed in the dogs given CS-CaCO_3_NP-DOX at a higher cumulative dose of above 200 mg/m^2^ concurs with the finding of Yang et al. [78] in the healthy rats and Maksimenko et al. [69] in mice since the concentration of DOX release from CS-CaCO_3_NP was very low to cumulatively elicit direct cellular injury.

Furthermore, the interstitial fibrosis observed in the myocardial tissue of the dogs given free DOX and higher dose of CS-CaCO_3_NP-DOX (at cumulative dose of 250 mg/m^2^) was in agreement with chronic model showing cardiomyocyte damage and fibrosis [79, 80], which could be as a result of cardiomyocyte compensatory action toward regeneration of myofiber undergoing degeneration or necrosis.

The kidney is a major excretory organ and is directly targeted by anthracycline, causing nephrotoxicity manifested as parenchyma and cellular damage [81]. However, the carrier molecules are entrapped within the slit diaphragm due to the size of the carrier molecules used in drug delivery leading to renal tubular tissue injury [82]. The glomerular and tubular damage observed in our study was similar to the lesion seen in rodent as reported by Cianciolo et al. [83] and Manno et al. [5] in rodent and mini pig animal models, respectively. In addition, our results also agree with the works of Anan et al. [84] as clusters of nuclear material were observed as a result of free radical production from lipid peroxidation.

Shivakumar et al. [85] reported that DOX has the potential to induce kidney tissue injury resulting from glomerulonephritis as observed in this study. The mechanism by which DOX induces nephrotoxicity shares similar pathway to that of cardiotoxicity which is induced through multifactorial pathways, ranging from oxidative stress, mitochondrial dysfunction, changes in transduction pathway [86]. One of the basic informative cellular changes observed due to DOX-induced damage is on the mitochondrial conformation as revealed by the ultrastructural examination of both cardiac muscle and renal tissue. Similarly, cardiorenal toxic effects were reported by Pereira et al. [87] and Berthiaume et al. [88] when repeated free DOX was administered, and myofibrillar disorientation and myocardial vacuolation with swallow to ruptured mitochondria were observed in cardiac tissue in the healthy animal model. However, interestingly, in this work, doses suggested for repeated dose administration of clinical dose regimens of CS-CaCO_3_NP-DOX 30 mg/m^2^ for 5 treatments did not witness any cardiorenal toxic effect resulting in cardiorenal structural and functional changes as compared to the dogs given an equivalent dose to free DOX 30 mg/m^2^.

The presence of adria cells observed in dogs was as a result of sarcoplasmic reticulum dilation as reported by Attia et al. [89] on the myocardial tissue damage. All the changes observed conform with the lesions observed histopathologically, on the light microscopes, and were similar to the previous reports in DOX-induced cardiotoxicity [89, 90].

The cardiac muscle of the dogs given a repeated dose of CS-CaCO_3_NP-DOX at a cumulative dose of 150 mg/m^2^ and at clinical regiment dose of 30 mg/m^2^ did not show any significant ultrastructural changes similar to the morphological architecture observed on the light microscopy. This was due to the small amount of DOX at sustained concentration released from the CS-CaCO_3_NP-DOX in normal physiological microenvironment as reported in the *in vitro* assay [18]. However, in contrary to the findings reported by Dhingra et al. [91] in mitochondrial signal, DOX induce necrosis in mice.

The kidney is also a targeted organ for DOX as previously reported by Salouege et al. [92] and Cianciolo et al. [83], but the order of the DOX organ affinity is less when compared to the heart due to cardiolipin concentration. Zhou et al. [93] demonstrated that the apical region of proximal tubules which have microvillus and abundant podocytes was responsible for the secretion of nephrin and podocin, which are structural components of slit diaphragm. The ultrastructural changes observed were similar to the changes reported by Ayla et al. [94] and Tao et al. [95] in their work, with clear visible vacuolization of the endothelial cells with thickening of the glomerular basement membrane and apoptosis of the podocyte foot processes. The degeneration of the foot process of the podocytes observed further confirms the glomerular obliteration of the corpuscular space observed on the same tissue in the histopathological analysis.

## 5. Conclusion

In conclusion, this study revealed that DOX-loaded cockleshell-derived CaCO_3_NP has less cardiotoxicity and nephropathic effects at a higher cumulative dose (250 mg/m^2^) as compared to the free DOX in healthy dogs. Thus, the study indicates that CS-CaCO_3_NP can minimize the off-target effects of the DOX in dogs with the aim of increasing quality of life in dogs that needs long-term therapeutic regime. This study further revealed the safety and tolerance of repeated dose administration of the CS-CaCO_3_NP-DOX in healthy dogs due to low amount of DOX exposure to vital organs, thus reducing the risk of cardiotoxicity and nephrotoxicity.

## Data Availability

The datasets generated and/or analysed during the current study are available from the corresponding author on reasonable request.
